# A Drug Screening Pipeline Using 2D and 3D Patient-Derived In Vitro Models for Pre-Clinical Analysis of Therapy Response in Glioblastoma

**DOI:** 10.3390/ijms22094322

**Published:** 2021-04-21

**Authors:** Sakthi Lenin, Elise Ponthier, Kaitlin G. Scheer, Erica C. F. Yeo, Melinda N. Tea, Lisa M. Ebert, Mariana Oksdath Mansilla, Santosh Poonnoose, Ulrich Baumgartner, Bryan W. Day, Rebecca J. Ormsby, Stuart M. Pitson, Guillermo A. Gomez

**Affiliations:** 1 Centre for Cancer Biology, SA Pathology and the University of South of Australia, Adelaide, SA 5000, Australia; Sakthi.Lenin@unisa.edu.au (S.L.); elise.ponthier@gmail.com (E.P.); kaitlin.scheer@mymail.unisa.edu.au (K.G.S.); erica.yeo@mymail.unisa.edu.au (E.C.F.Y.); melinda.tea@unisa.edu.au (M.N.T.); Lisa.Ebert@sa.gov.au (L.M.E.); mariana.om8@gmail.com (M.O.M.); Stuart.Pitson@unisa.edu.au (S.M.P.); 2 Adelaide Medical School, University of Adelaide, Adelaide, SA 5000, Australia; 3 Cancer Clinical Trials Unit, Royal Adelaide Hospital, Adelaide, SA 5000, Australia; 4 Flinders Health and Medical Research Institute, College of Medicine & Public Health, Flinders University, Adelaide, SA 5042, Australia; santoshpoonnoose@me.com (S.P.); rebecca.ormsby@flinders.edu.au (R.J.O.); 5 Department of Neurosurgery, Flinders Medical Centre, Adelaide, SA 5042, Australia; 6 Cell and Molecular Biology Department, Sid Faithfull Brain Cancer Laboratory, QIMR Berghofer Medical Research Institute, Brisbane, QLD 4006, Australia; Ulrich.Baumgartner@qimrberghofer.edu.au (U.B.); bryan.day@qimrberghofer.edu.au (B.W.D.); 7 Faculty of Health, Queensland University of Technology, Brisbane, QLD 4006, Australia; 8 Faculty of Medicine, University of Queensland, Brisbane, QLD 4072, Australia

**Keywords:** glioblastoma, organoids, personalized medicine, therapy resistance, drug screening, tumor microenvironment

## Abstract

Glioblastoma is one of the most common and lethal types of primary brain tumor. Despite aggressive treatment with chemotherapy and radiotherapy, tumor recurrence within 6–9 months is common. To overcome this, more effective therapies targeting cancer cell stemness, invasion, metabolism, cell death resistance and the interactions of tumor cells with their surrounding microenvironment are required. In this study, we performed a systematic review of the molecular mechanisms that drive glioblastoma progression, which led to the identification of 65 drugs/inhibitors that we screened for their efficacy to kill patient-derived glioma stem cells in two dimensional (2D) cultures and patient-derived three dimensional (3D) glioblastoma explant organoids (GBOs). From the screening, we found a group of drugs that presented different selectivity on different patient-derived in vitro models. Moreover, we found that Costunolide, a TERT inhibitor, was effective in reducing the cell viability in vitro of both primary tumor models as well as tumor models pre-treated with chemotherapy and radiotherapy. These results present a novel workflow for screening a relatively large groups of drugs, whose results could lead to the identification of more personalized and effective treatment for recurrent glioblastoma.

## 1. Introduction

Glioblastoma is the most common and aggressive form of primary brain tumor of the central nervous system (CNS) in adults and is responsible for 80% of all malignant primary tumors of the brain [1]. Its worldwide incidence rate is 3 per 100,000 people [2,3], and it is associated with an extremely poor prognosis (median survival <16.8 months [4] and five-year survival rate <5.2% post diagnosis [5,6]). The current standard treatment for glioblastoma patients is maximum surgical removal of the tumor, followed by radiotherapy and chemotherapy, frequently with temozolomide (TMZ) [5,6]. Despite these treatments, recurrence of glioblastoma within 6–9 months of initial diagnosis is almost inevitable [7], for which there are no standard therapies available [8].

The poor prognosis in glioblastoma is at least partly attributed to the high level of inter- and intra-tumoral heterogeneity [9]. Inter-tumoral heterogeneity of glioblastoma was originally identified and categorized through transcriptional profiling studies into four distinct molecular subtypes: pro-neural, mesenchymal, classical, and neural [10,11,12]. Classical subtypes are characterized by genetic alteration of genes such as *EGFR*, *TP53* and *CDKN2A*. Proneural subtypes usually contain genetic alterations in *PDGFRA* and *IDH* [10,11]. Mesenchymal subtypes exhibit dysregulated expression of *YKL40*, *VEGF* and *MET* genes and *NF1/PTEN* co-mutation, which are associated with epithelial-to-mesenchymal transition (EMT) [13]. Proneural and mesenchymal expression subtypes are mostly associated with poor prognostic outcome and poor survival rate [14]. The neural subtype was characterized by the expression of neuronal markers such as *SLC12A5*, *GABRA1*, *SYT1* and *NEEL* [10,11,12], but this subtype was reported later to be non-tumor specific and the result from contamination of normal cells [15].

In addition to inter-tumor heterogeneity, there is a significant level of intra-tumoral heterogeneity in glioblastoma [9]. This relates to the presence of distinct cancer cell subclones within a single tumor [16]. Several studies have investigated the intra-tumor heterogeneity at the genomic level, with a number of these identifying differential expression of receptor tyrosine kinases (RTKs) in different cancer cell populations [17,18]. Three different RTKs—epidermal growth factor receptor (EGFR), mesenchymal-to-epithelial transition (MET) and platelet-derived growth factor receptor alpha (PDGFRα)—demonstrate highly variable gene expression in individual tumor cells [17,19]. Heterogeneous expression of several other common genes, which include isocitrate dehydrogenase (*IDH1*), telomerase reverse transcriptase (*TERT*), phosphatase and tensin homologue (*PTEN*), neurofibromatosis type 1 (*NF1*) gene and O^6^-methylguanine-DNA methyltransferase (*MGMT*), have also been described within glioblastoma tumors [9].

In addition, at the cellular level, glioblastoma intra-tumor heterogeneity is characterized by variable gene expression for a number of different transcriptional programs as determined in single cell RNA sequencing (scRNAseq) experiments, including oncogenic signaling, proliferation, immune response and hypoxia [20,21,22,23,24,25,26]. This intra-tumor heterogeneity can be explained by the existence of glioma stem cells (GSCs), also termed as tumor initiating cells [27], that present stem cell properties (stemness), which confers on these cells the capabilities of self-renewal and multi-linage differentiation [28]. Thus, GSCs contribute to cellular heterogeneity in a hierarchical fashion of differentiation by interconverting into a wide range of distinct subpopulations of tumor cells within an individual tumor mass [29], where these subpopulations promote different aspects of tumor biology, including growth, invasion and the development of therapy resistance [30]. The tumor plasticity conferred by the presence of stem-cell-like properties is a determinant in the response of tumor cells to microenvironmental signals [31,32], constituting a bidirectional process in which the phenotypic shift between GSCs and other differentiated tumor cell types occurs due to selective pressures including cell–matrix and cell–cell interactions, environmental factors (metabolism, hypoxia and ECM) and drug therapies [28,33].

Despite the above-mentioned advances in our understanding of the genetic make-up and inter- and intra-tumoral heterogeneity in glioblastoma, treatment protocols for newly diagnosed glioblastoma patients in the clinic have not substantially changed in the last 15 years [34]. This still consists of maximal surgical resection of the tumor followed by TMZ chemotherapy and radiotherapy [35,36]. Apart from TMZ, there are four other drugs that have been approved by FDA for glioblastoma treatment; however, they provide limited benefit to patients [37]. This reveals an urgent need for the development of better preclinical tools that facilitate rapid and efficient screening of new drugs that can then be used in the clinic. Several pre-clinical models such as cell lines, tissue culture and mouse models have been designed to test and evaluate the efficacy of drug therapies to successfully translate into clinical trials in glioblastoma patients. However, these frequently fail to accurately recapitulate the biology of glioblastoma tumors in humans [38,39,40]. For example, traditional two-dimensional (2D) culture of immortalized cell lines lacks the capabilities to replicate important features of primary tumors such as stemness, genetic heterogeneity and tumor microenvironment [41]. Three dimensional (3D) tumor-sphere models generated from glioma stem cells lack the ability to interact with the extracellular matrix components and cells present in the tumor microenvironment [42]. Finally, animal models fail to completely capture anti-tumor immune responses in humans due to the use of immunodeficient mice [35,43].

**Figure 1:**
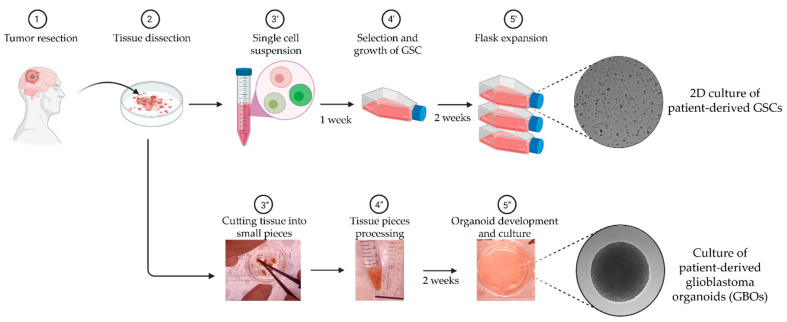
Patient derived in vitro models of glioblastoma used in this study. Following surgical resection, the tissue sample is dissected and streamed into two workflows: (′) the generation of two dimensional (2D) culture of low passage patient-derived glioma stem cells (GSCs); and (″) the culture of three dimensional (3D) patient-derived glioblastoma explant organoids (GBOs). Key steps (3–5) for each workflow are mentioned and the gray-background images correspond to DIC microscopy images.

Therefore, to overcome the limitations mentioned above, a suitable in vitro model that closely represents patient glioblastoma tumors is required [38]. Aligned to this concept are the recently adopted use of low passage patient-derived GSCs and organoids (Figure 1). GSCs recapitulate the intratumor cancer cell heterogeneity and reflect cancer stem cell properties of the primary tumor [44,45,46]. Xenograft tumors generated through the injection of patient-derived GSCs are highly invasive and display the key hallmarks of glioblastoma seen in patients, namely hypercellularity, nuclear atypia and the presence of mitotic figures, with or without microvascular proliferation [46]. In addition, these cell lines that more accurately reflect the biology of GSC within the tumor, are more clinically relevant compared to high-passage/commercially available cell lines and are also ideally suitable for high-throughput personalized screening of new therapeutic drugs [46].

In addition to GSCs, patient-derived glioblastoma explant organoids (GBOs) have recently emerged as a promising model for studying glioblastoma tumor cells within a more physiologically relevant tumor microenvironment [9,38,47] (Figure 1). GBOs are produced by culturing glioblastoma tumor tissue pieces of ~1 mm diameter in a defined media in the absence of Matrigel [48,49]. GBOs retain the cytoarchitecture and tumor–stroma interactions of the original glioblastoma tumor [49] as well as its inter- and intra-tumor heterogeneity, retaining important tumor microenvironmental characteristics that include microvasculature, immune cell populations and hypoxia gradients. At the cellular level, gene expression of tumor cells and non-malignant cells such as macrophages/microglia, T-cells and myelinating oligodendrocytes are also similar to the corresponding original tumors. Finally, and very importantly, the therapeutic responses of GBOs generated from different tumors to chemotherapy, radiation and chimeric antigen receptor (CAR)-T cell treatments vary depending on the genetic alterations that were present in the original tumors [49].

Patient-derived models have thus emerged as promising pre-clinical platforms for testing glioblastoma chemotherapeutics. Here, we propose a pipeline that combines screening in both 2D low passage patient-derived GSC and 3D GBOs, for screening of drugs (U.S. Food & Drug Administration (FDA)- approved, in Phase II–IV glioma/brain tumor clinical trials (accessed in April 2020) or under investigation) that target different hallmarks of glioblastoma. We believe that this approach could: (i) accelerate the implementation of personalized treatments for glioblastoma in the clinical setting; (ii) overcome current limitations for developing and evaluating the efficacy and safety of new drugs [50]; and (iii) expedite the drug repurposing process for glioblastoma [51].

## 2. Results

### 2.1. Selection of Drugs for Screening in Patient-Derived In Vitro Models

**Figure 2:**
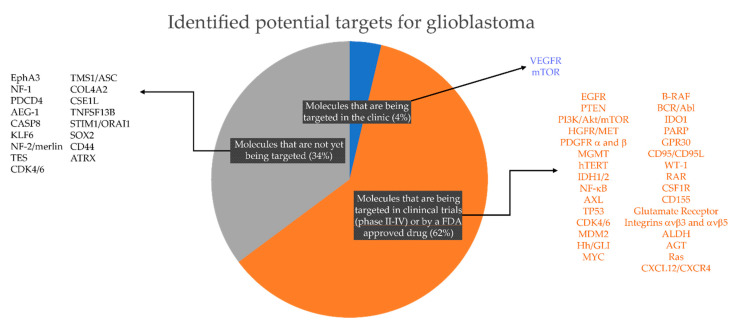
Potential targets for glioblastoma based on literature review, current clinical management and clinical trials for glioblastoma. Note that there are only 2 (4% of the total number of targets identified in this study) targeted therapies currently approved for glioblastoma treatment in the clinic; 31 targeted therapies (62% of total) for which there is a U.S. Food & Drug Administration (FDA)- approved drug and/or are currently being evaluated in glioma/brain tumors clinical trials (https://clinicaltrials.gov); and 17 (34%) recently identified molecular targets for which there is an available drug either FDA-approved but not listed for glioblastoma or an inhibitor developed but not yet clinically approved for any medical condition.

Notably, most of the efforts thus far in drug screenings and clinical trials have focused either on targeting tumor cell proliferation or using commercially available drug libraries that contain multiple drugs for a single target. As a result, although these libraries are made of a few thousand compounds, they are not equally distributed across the targets (e.g., Selleck Chemicals and MedChemExpress compound libraries). In addition, while glioblastoma tumors are highly heterogeneous, it is likely that glioblastoma patients will be classified, based on their response to different treatments and metadata information, only into a small number of “responder” groups. For example, thus far only three tumor subtypes, four cancer stem cell transcriptional states and a few biomarker-based predictors of therapy response (*MGMT* methylation and *IDH1* mutation) have been described [37]. Thus, it is possible that using patient-derived in vitro models to screen a small and carefully selected list of compounds that inhibit key molecular targets across different glioblastoma subtypes can increase feasibility and efficiency for its rapid implementation in precision neuro-oncology [37]. With this in mind, we first focused on the identification of molecular targets that contribute to different biological processes in glioblastoma (invasion, cell death resistance, transition between transcriptional states, cell–ECM adhesion, cell–cell adhesion, tumor metabolism, etc.) and are also expressed in different cancer stem cell populations (mesenchymal, oligodendrocyte-like, astrocyte-like, and neuronal precursor-like [24]). We identified 2 molecular targets that are currently being targeted in the clinic, 31 molecular targets that are in Phase II–IV clinical trials and 17 molecular targets that have been recently discovered as new potential targets for glioblastoma (Figure 2).

From the list of molecular targets, we then searched for the corresponding drug inhibitors that are suitable for pre-clinical screening (Table 1) also considering the potential for direct clinical translation of the screening results. We thus focused on inhibitors that are: (i) currently in clinical use or Phase II–IV clinical trial for glioblastoma (best inhibitor available for each target, clinical trial still ongoing and no dose-limiting toxicities reported) (34 inhibitors, including 5 FDA approved drugs); (ii) FDA approved drugs for medical conditions distinct from glioblastoma and currently not listed in clinical trials for glioblastoma (14 inhibitors); and (iii) inhibitors selected from current literature and which have been shown to block specific signaling pathways in glioblastoma cells but which are as yet neither targeted in clinical trials nor by FDA approved drugs (16 inhibitors).

### 2.2. Drug Screening Pipeline

In vitro patient-derived models have their own advantages and limitations [38]. As mentioned above, 2D cultures of low-passage patient-derived GSCs are good models that replicate the genetic makeup of the tumors in the patient and are capable of exhibiting plasticity in response to changes in the microenvironment [46,114,115]. However, these models lack the stromal component of the tumor mass and are poor at recapitulating tumor–stroma interactions. Moreover, culture conditions used to grow GSCs can also impact the way they respond to treatment [116]. On the other hand, GBOs have now emerged as a better in vitro model that recapitulate several key aspects of the patient’s tumor, in particular the presence of different cancer cell types and cells in the microenvironment including microglia, endothelial cells, pericytes and T-cells (see also [117] for a historic perspective of patient-derived glioma tissue explant cultures). Nonetheless, the methods for processing tumor biopsies and the culture of GBOs are low-throughput and less amenable to fully automated procedures (i.e., use of liquid handler robots, automated pipetting, automated high-resolution imaging) when compared to 2D cultures of patient-derived GSCs. This limitation is compounded by the reduced availability of assays that permit rapid and reliable measurement of GBO responses to drug treatment in a high-throughput screening setup.

**Figure 3:**
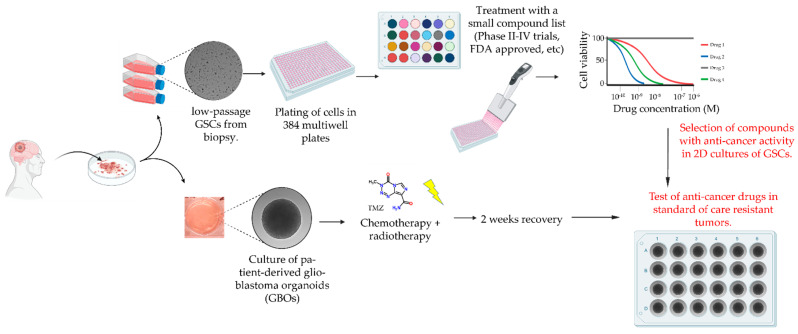
Drug screening pipeline in matched 2D and 3D patient-derived in vitro models for glioblastoma.

To take advantage of each of the models and maximize throughput, speed and scalability of the drug-response assay while simultaneously overcoming their inherent limitations, we developed a new drug screening pipeline that can deliver rapid results within a clinically relevant time frame (Figure 3). This pipeline includes measurement of the IC_50_ response in 2D GSC cultures for each of the drugs in Table 1 to identify candidates that can then be tested in matched 3D GBO cultures pre-treated with standard chemotherapy and radiotherapy.

### 2.3. Drug Screening in 2D Cultures of Patient-Derived GSCs

To determine the effectiveness of each of the selected drugs to alter the viability of glioblastoma cells, we performed our drug screening on two patient derived primary glioma stem cell lines: FPW1 [44,46,118] and G18-T (Table 2) [46].

**Figure 4:**
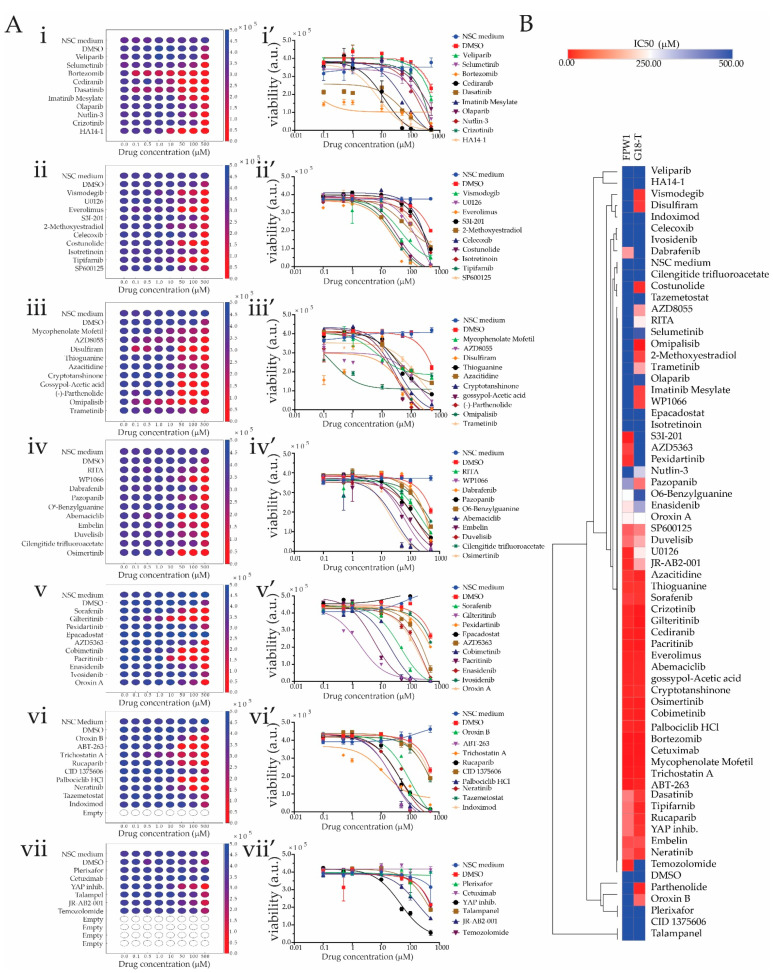
Drug screening using patient-derived 2D GSC cultures. (**A**) (**i**–**vii**) Heatmaps of each plate representing cell viability results for G18-T cells treated with eight drug concentrations for each drug as well as negative control (StemPro Neural Stem Cell serum-free medium, NSC Medium) and vehicle control (dimethyl sulfoxide, DMSO) for each plate. The color bar of each heatmap shows bioluminescent units, an index of the number of viable cells. Red indicates low cell viability index and dark blue indicates high cell viability index. (**i’**–**vii’**) Dose vs. Response graphs for the test conditions as in (**i**–**vii**). Data are mean ± standard error of mean (SEM) for an experiment performed in quadruplicate. (**B**) Heatmap with hierarchical clustering representing the IC_50_ of each drug in FPW1 and G18-T cells. Bright red indicates low IC_50_ and bright blue indicates high IC_50_.

G18-T was selected as this GSC line also had a matched GBO culture derived from the same patient biopsy tumor tissue. FPW1, which has been previously described [46], was selected as this GSC line has an unmethylated *MGMT* promoter, a status which is linked to TMZ resistance in glioblastoma [119] and serves us as a suitable model to test whether the selected drugs are effective at altering the viability of this cell line. A cell viability assay was executed in 384-well multiwell format using CellTiter-Glo^®^ 2.0, which is a bioluminescence assay designed to detect cellular metabolic adenosine triphosphate (ATP) levels within viable cells. This assay was performed after a 72 h treatment of cells with drugs/inhibitors, with treatment starting 24 h after cell plating in 384-well plates. All procedures including drug dilutions, drug and reagent additions to cells and mixing were performed using automated liquid handling robot (Opentrons OT-2) with minimal manual intervention. In total, 65 drugs/inhibitors (64 listed in Table 1 + temozolomide (TMZ)) were tested in quadruplicate at eight different drug concentrations, 500, 100, 50, 10, 1, 0.1 and 0 µM, with additional control conditions including vehicle only (dimethyl sulfoxide, DMSO) and culture media without both vehicle and drug (StemPro Neural Stem Cell serum-free medium, NSC medium). Bioluminescent data were then averaged across replicates and plotted both as a heatmap to visually assess drug responses and dose–response curves for calculation of drug IC50 values for each 384-well plate (Figure 4 and Figure S1 show the results for G18T and FPW1 lines, respectively). 

From the cell viability assay results, 16 drugs were found to be ineffective against G18-T tumor cells. For three (Epacadostat, Plerixafor and Cetuximab) of these 16 drugs, cell viability was not altered, whereas, for the remaining 13 drugs (Veliparib, Selumetinib, Crizotinib, S3I-201, Cilengitide trifluoroacetate, Pexidartinib, Ivosidenib, CID 1375060, Tazemetostat, Indoximod, Talampanel, JR-AB2-001 and Temozolomide), it was reduced only at the highest drug concentration (500 µM), an effect that was attributed to the presence of DMSO as a similar pattern was observed with DMSO-only control (Figure 4A). Eight drugs [Selumertinib, Dasatinib and HA14-1 (Figure 4A(i,i’)); AZD8055, Disulfiram and Omipalisib (Figure 4A(iii,iii’)); Gilteritinib (Figure 4A(v,v’)); and Trichostatin A (Figure 4A(vi,vi’))] were observed to reduce GSC viability at 0.1 µM concentration. This was further revealed in the dose vs. response graphs, where the cell viability of G18-T cells started to decline from 0.1 µM concentration and reached minimal cell viability at approximately 100 µM concentration (Figure 4A(i’,iii’,v’,vi’)). The remaining 41 drugs generated a moderate response against G18-T tumor cells with the ability to reduce cell viability in G18-T tumor cells at the three highest concentrations (50, 100 and 500 µM), as evident in both the heatmaps and the dose vs. response curves (Figure 4A). Overall, these results suggest that 8 (12%) of the tested drugs exhibited a strong response at low concentration, 41 drugs (63%) exhibited a moderate response and 16 drugs (25%) were ineffective against G18-T tumor cells. Thus, approximately 70% of selected drugs with specific molecular targets altered G18-T GSC cell viability.

To compare drug responses across patients, we performed IC_50_ calculations for each drug in both of the GSC lines using non-linear regression analysis to identify groups of compounds that were either effective in reducing cell viability in one or both patient-derived GSC lines (Figure 4B). The IC_50_ for each drug was calculated and the numerical values plotted as a heatmap with hierarchical clustering to rapidly identify drugs with similar and dissimilar responses across the patient-derived samples. From the heatmap representation, we identified three different group of drugs based on their differential effect on cell viability of the two patient-derived cell lines analyzed (Figure 4B). The first group contained 29 drugs that had an inhibitory effect on cell viability of both cell lines with IC_50_s in the range of 0.1–250 µM (Figure 4B). The second group contained 18 drugs that exhibited little to no effect on either cell line, with an IC_50_ range of 250–500 µM (Figure 4B). Finally, the third group exhibited a selective effect either on G18-T or FPW1 patient-derived GSC lines with 13 drugs having an inhibitory effect on G18-T cells only and 5 drugs having an inhibitory effect on FPW1 cells only (Figure 4B). This variable response suggests the presence of orthogonal mechanisms that support viability in the patient-derived GSC lines, which could be targeted selectively by using the panel of drugs listed in Table 1. Further inspection of the identity of the drug targets supported this notion and revealed that most of the targets whose corresponding drug presented selective effects on viability on each of these cell lines (i.e., Group 3) were non-overlapping (Table 3). Of the drugs which selectively targeted the G18-T cells, five drugs presented very strong effects on GSC viability (IC_50_ in the range 50 µM): Vismodegib, Disulfiram, Parthenolide, Omipalisib and Costunolide (Figure 4B).

We also found that TMZ, which is the standard treatment for glioblastoma, did not alter G18-T cell viability (Figure 4A). When we compared the IC_50_ of TMZ in G18-T and FPW1 cells, we found that TMZ had a selective inhibitory effect on FPW1 cells (Figure 4B). It was predicted that FPW1 would be resistant to TMZ as this cell line was derived from a biopsy with unmethylated status of the *MGMT* promoter; however, in our hands, the G18-T line also behaved as a TMZ-resistant model. The mechanism by which this cell is resistant is not yet known as there were no data available to us on MGMT methylation status and/or genetic mutation profile for this GSC line at the moment of performing these studies.

Overall, the finding from this drug screening analysis conducted on patient-derived GSCs revealed different types of drug responses, a finding that led to us to further investigate the response of G18-T [TMZ+radiation] resistant cells to the addition of the panel of drugs that we found selectively alter this cell line viability.

### 2.4. Effect of Drug Treatment in 2D and 3D In Vitro Models of Primary and Standard of Care Resistant Glioblastoma

#### 2.4.1. Generation of TMZ+radiation (“Stupp”) Resistant 2D and 3D Patient-Derived In Vitro Models

**Figure 5:**
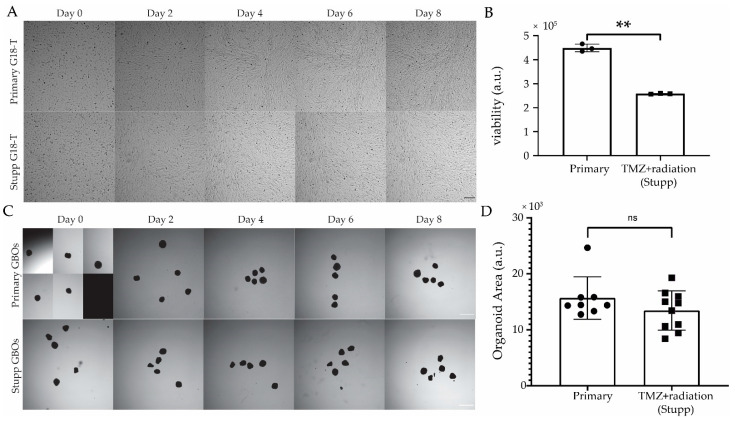
Resistance to standard of care treatment “in a dish” in 2D cultures of GSC and in GBOs. (**A**) Brightfield images of primary G18-T (untreated) and Stupp G18-T (temozolomide (TMZ)+radiation treated) 2D cell cultures over the 8-day treatment period. Day 0 images were taken immediately before treatment (Scale bar 300 µm). (**B**) Cell viability measured at the end of the protocol using CellTiter-Glo^®^ 2.0 bioluminescence assay. (**C**) Brightfield images of Primary GBOs (untreated) and Stupp GBOs (TMZ+radiation treated) over the 10-day treatment period, with Day 0 images taken immediately before treatment (Scale bar 1 mm). (**D**) 2D area of GBOs at Day 10 for each treatment condition. All images were processed using ImageJ with consistent settings applied for all images. Data are mean ± SEM, *n* = 4 (B), *n* = 10 (**D**); **, *p* < 0.01; ns, not significant, two tailed, *t*-test.

As TMZ was found to be ineffective against G18-T cells, we decided to evaluate whether this cell line and its matched GBO were resistant to TMZ when administered in conjunction with radiation (i.e., standard of care or Stupp protocol [5]). For this, we conducted chemo (TMZ) and radio (10 Gy) therapy on patient-derived in vitro models as we have done previously [114]. For this, we used GBOs that were 10 weeks old with an average diameter of 0.53 mm. “Stupp” G18-T cells and GBOs (*n* = 10 GBOs) received treatment with both TMZ (50 µM) and irradiation (2 Gy) every 2 days over a 10-day period. “Primary” G18-T cells and GBOs (*n* = 10 GBOs) were cultured in normal growth medium without TMZ and were not irradiated. Stupp and primary GSC (or GBOs) were cultured in the same 6-well plate during this protocol to control for any effect caused by removing cells from the incubator during drug and irradiation treatments. Images were taken using an InCell Analyser 2200 high-content microscope every 2 days to measure changes in cell confluency of 2D GSC cultures (Figure 5A,B) and in size of GBOs (Figure 5C,D). From the images of 2D cultures (Figure 5A), there was no significant reduction in cell viability associated with the treatment with TMZ+radiation. However, we observed a reduction in the growth rate and cell viability of Stupp G18-T cells when compared to primary G18-T cells (Figure 5A,B).

A similar assessment of the representative images from each treatment group of GBOs (Figure 5C,D) revealed no observable effect of the Stupp protocol on GBOs at any of the timepoints to the end of treatment (Day 10) or in comparison to the primary GBOs. There were no significant changes in GBO size, as measured by 2D area for either group over the treatment period, suggesting that TMZ plus radiation treatment was also ineffective in causing changes in the viability of GBOs. This suggests that both G18-T cells and the corresponding GBOs resisted to the current standard of care. These patient-derived models were allowed to recover for two weeks (to model glioblastoma treatment in the clinic) and were then used for further evaluation of those drugs which exhibited selective response towards untreated G18-T cells but not FPW1 cells.

#### 2.4.2. Response of Stupp Treated G18-T Cell Line and GBO to Selected Drugs

**Figure 6:**
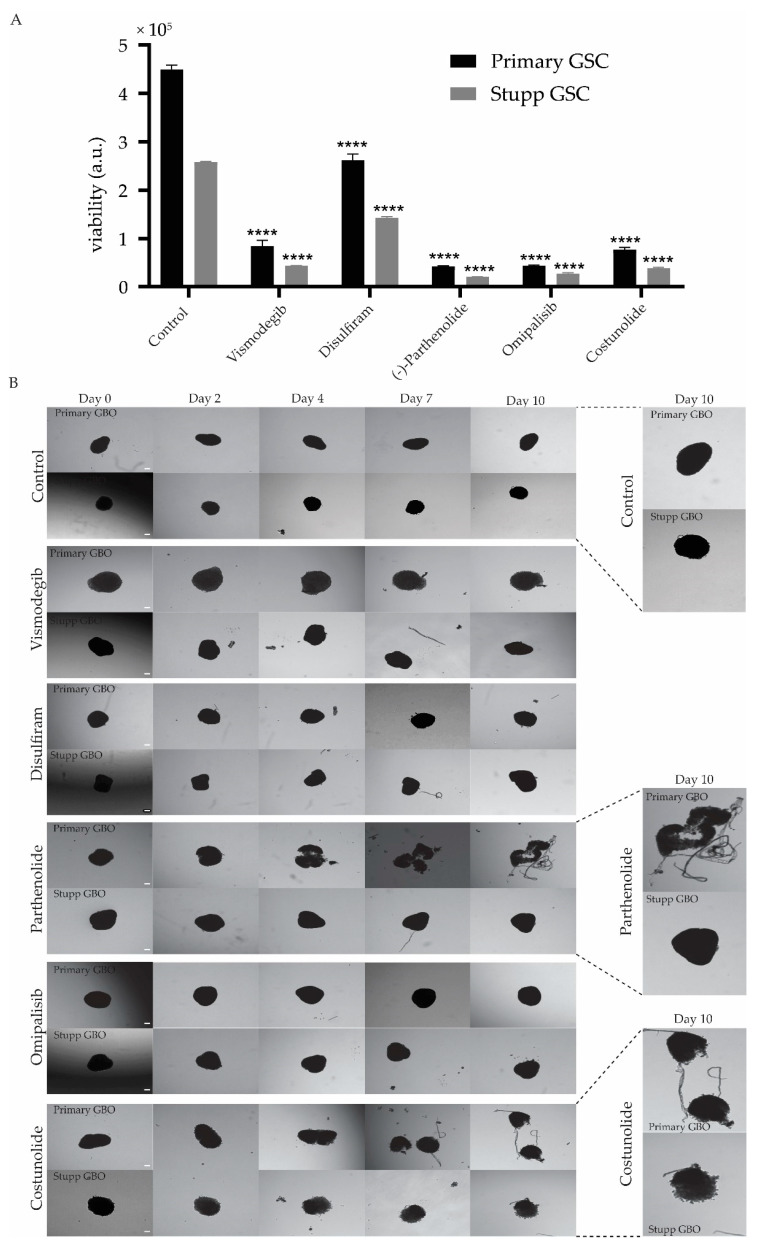
Response to second line treatment of treatment naïve and resistant glioblastoma. (**A**) Primary and Stupp G18-T cell line response to the addition of Vismodegib, Disulfiram, Parthenolide, Omipalisib or Costunolide. Data represent cell viability for each treatment group with the addition of each of the five selected drugs. Data are mean ± SEM for four replicates. All treatment groups were compared with their respective group (primary or Stupp) control (**** *p* < 0.0001, TWO-WAY ANOVA with Sidak correction for multiple comparisons). (**B**) GBO response to the addition of selected drugs. Brightfield images of primary GBO (untreated) and Stupp GBO (TMZ+radiation treated) with additional treatment with each selected drug over the 10-day treatment period, with Day 0 images taken before the start of the treatment. Scale bar 0.1 mm. Magnified images of primary and Stupp GBO for Control, Parthenolide and Costunolide treatment are also presented.

GBOs and G18-T 2D cell cultures that had previously been exposed to either the control or Stupp conditions, were treated with 50 µM of Vismodegib, Disulfiram, Parthenolide, Omipalisib or Costunolide (Figure 6). This drug concentration was well below the IC_50_ value determined for each drug in the G18-T cell line (as shown in Figure 4). 

A cell viability assay was performed after five days of drug treatment to evaluate the effectiveness of each drug on the primary and Stupp treated GSCs in 2D culture. It is evident that all five drugs were effective in reducing cell viability, to varying extents, both primary and Stupp resistant G18-T cells (Figure 6A). Disulfiram had the least effect on G18-T cell viability compared to the other four drugs. Costunolide and Vismodegib had similar effectiveness and Omipalisib and Parthenolide demonstrated the greatest effect on cell viability. Overall, these drugs proved to be significantly more effective at reducing viability in cells previously treated with TMZ + radiation.

In contrast primary and Stupp GBOs treated with Vismodegib or Disulfiram showed no clear response, although the edges of the GBOs seemed to be altered slightly compared to the control primary GBO (Figure 6B), suggesting that these treatments were ineffective in killing both primary and Stupp resistant GBOs. Omipalisib had no effect on the GBOs as there were changes neither in primary or Stupp GBO (Figure 6B). Parthenolide and Costunolide were the only drugs that clearly impacted GBOs, with Parthenolide causing dissociation of the primary GBO into fragments (Figure 6B). However, Parthenolide had no effect on Stupp treated GBOs (Figure 6B). This suggests that Parthenolide was effective when administrated alone but ineffective when administered after TMZ+radiation treatment. Costunolide appeared to have an impact on both primary and Stupp GBOs as it caused the dissociation of primary GBO tissue and affected the edge morphology in Stupp GBOs (Figure 6B). These alterations seemed to occur early after treatment and became evident from Day 3 (Figure 6B).

Overall, these results indicate that the selected five drugs were particularly effective against TMZ+radiation treated G18-T cells. However, Costunolide was the only drug that showed efficacy on primary and Stupp GBOs.

## 3. Discussion

This study analyzed the effectiveness of targeted inhibitors using patient-derived in vitro models of glioblastoma. Our main goal was to select a repertoire of inhibitors/drugs that target a diverse set of cellular processes that contribute to glioblastoma aggressiveness (proliferation, invasion, stem cell properties, resistance to cell death, metabolism, etc.). We aimed to test the efficacy of these inhibitors using patient-derived in vitro models that better recapitulate inter- and intra-tumor heterogeneity, as well as the response to treatment [9,37]. This is an important consideration for the clinical management of glioblastoma as there are currently only four possible treatments that target tumor cell proliferation and angiogenesis [9,37], which provide limited benefits to patients [120]. Moreover, we restricted our analysis to inhibitors/drugs (majority small molecules) that are either FDA approved (i.e., can be repurposed to glioblastoma) or in Phase II–IV clinical trials (i.e., already passed the safety test and have shown either a favorable or heterogeneous response in patients). We believe this approach has the potential to be rapidly implemented in the clinic since it has the benefit of having a predictive capacity for identifying targets that are relevant and the most effective for each individual patient, which will be a game changing situation in the clinical management of glioblastoma.

Furthermore, we showed that this small group of inhibitors can be used as a platform for the screening and identification of key molecular targets that contribute to the overall viability of patient-derived tumor cells (in either 2D or 3D models) and used these models to test the efficacy of candidate compounds on patient-derived tumor samples that had been subjected “in a dish” to the standard of care treatment for glioblastoma (TMZ+radiation). This approach thus has the benefit of providing information that is relevant for the treatment of Stupp resistant tumors and the clinical management of recurrent glioblastoma. Below, we discuss different aspects of our workflow and key findings to date, based on the analysis of two patient-derived glioblastoma stem cell 2D cultures and one matched glioblastoma explant organoid culture.

### 3.1. Drug Screening Using a Combination of 2D and 3D Patient-Derived In Vitro Models for Glioblastoma

In this work, we optimized a workflow using automated liquid handling (Opentrons OT-2) that allowed us to perform high-throughput drug screening on patient-derived glioma stem cell 2D cultures to narrow down a group of selective drugs with the potential to target cancer cells, which then could be tested in a more complex patient-derived in vitro model GBO, which better recapitulate the histology and microenvironment composition of the tumor. This allowed us to perform in an automated manner a total of 4288 tests for both G18-T and FPW1 patient-derived GSCs, reducing the chances of technical errors associated to manual handling. Using 2D cultures to narrow down the list of targets expedites the whole process as 2D assays are readily miniaturizable (e.g., 96- and 384-well format) and not cell passaging, media change or cell/supernatant harvesting is required from the moment cells are plated in multiwell plates (Day 0) until bioluminescence is measured (Day 3). Using this approach, we were able to identify potential drug candidates from 2D cultures of patient-derived GSC within a week, which adds to the two weeks needed to establish GSC 2D cultures and GBOs, an overall short time-frame for obtaining clinical relevant results. For this, we also complemented our approach with custom made software scripts that allowed us to extract the data and graphically represent it (Supplementary Materials). In the future, is likely that such large-scale screening will be feasible with patient-derived 3D tumor organoids, but currently the time required for expansion of such a large number of organoids and the yield of organoids per sample is restrictive [37,48,49]. Complementary 2D and 3D screening thus provides an extra advantage as the initial screen using a 2D culture of patient-derived GSCs enabled us to narrow down the group of selective drugs with potential to kill cancer cells from a particular patient. This smaller group of drugs can then be assessed in a more complex 3D glioblastoma organoid model that better recapitulates the composition of the tumor microenvironment. We envisage this approach to be useful as an entry level drug screening of patient-derived samples in the diagnostic setting and in clinical trials.

### 3.2. Drug Inhibitors Have Varying Effects on Different Patient-Derived GSC Cultures

While patients have different responses to treatment in the clinic, this observation has not been extensively characterized in patient-derived GSCs [46]. Until now, studies have only examined the sensitivities of patient-derived in vitro models to standard of care treatment [46]. Thus, we decided to test a panel of drugs on two patient-derived glioma stem cell 2D cultures for their ability to inhibit the growth and/or inhibit tumor cell viability. The FPW1 cell line has been well characterized [114], whereas G18-T was recently derived at our institute from resected patient tissue.

Analysis of the cell line responses permitted us to categorize the drugs into three different groups based on their differential response to drug treatments (Figure 4B and Table 3). This correlates with the fact that patient-derived samples might have different genetic and transcriptional make up, which is expected due to the high level of inter tumoral heterogeneity in glioblastoma [46,114,118,121,122]. Therefore, patient-derived GSCs that exhibited selective responses to specific drugs probably had higher expression levels of the corresponding target molecules and/or are intrinsically more dependent on the activity of such targets. This variation in the drug responses between the two patient-derived GSCs in 2D culture is evidence of the heterogeneity of glioblastoma and also reflects the variation in response to treatment between patients observed in the clinic [123]. We expect that the application of this screening approach to a larger panel of well characterized 2D cultures of patient-derived GSCs will permit us to identify correlations between drug response and genotype (*IDH* mutation, *EGFR* amplification, *PTEN* mutation, etc.) [46].

### 3.3. Response of Stupp Treated 2D and 3D Cultures to Vismodegib, Disulfiram, Parthenolide, Omipalisib and Costunolide

Intratumoral heterogeneity of glioblastoma is a major contributing factor to therapy resistance. Under treatment conditions, tumor cells have the capacity to switch from one state to an alternative state that allows them to adapt and become resistant to treatment [24]. To successfully address this issue, given the context of tumor recurrence in the clinic, we treated the patient-derived in vitro models with TMZ+radiation (10 days), followed by two-week recovery and further treatment with drugs that showed selective efficacy in the corresponding GSC 2D culture. The two-week recovery period was introduced to mimic at some extent the management of glioblastoma in the clinic, where recurrence occurs almost in every case of glioblastoma and patients are left untreated after chemoradiotherapy until recurrence is detected. Drugs that showed selective efficacy in the corresponding GSC 2D culture (in this case Vismodegib, Disulfiram, Parthenolide, Omipalisib and Costunolide) were chosen because these drugs may present less cytotoxic effects compared to other drugs that broadly affect cell viability of GSCs that were derived from different patients.

We found that TMZ+radiation treatment followed by Parthenolide~Omipalisib > Vismodeib ~ Costunolide > Disulfiram was more effective at reducing G18-T cell viability in 2D cultures than TMZ+radiation or each of these drugs alone. In contrast, analysis of matched GBOs suggested that GBOs were not affected by the first treatment of TMZ+radiation and, unlike the 2D cultures, the treatment with Vismodeib, Disulfiram or Omipalisib were not effective in Stupp resistant GBOs. This could be because the outer region GBOs were mainly comprised of rapidly proliferating cells which were constantly exposed to the medium containing the drugs. TMZ+radiation followed by treatment with Parthenolide also did not have any effect on GBOs, however, treatment with Parthenolide on primary GBOs caused dissociation of the GBOs, suggesting that TMZ+radiation treatment alter the GBOs dependency Parthenolide molecular targets (i.e., NF-κB, Table 1). The most effective treatment was with Costunolide, which caused deterioration of both primary and Stupp resistant GBOs. Costunolide is a sesquiterpene lactone inhibitor of telomerase reverse transcriptase (TERT) with reported antioxidative, anti-inflammatory, antiallergic, neuroprotective and anticancer properties [124]. Past studies showed that costunolide treatment reduces human TERT (hTERT) telomerase activity by downregulation of hTERT mRNA, resulting in cell cycle arrest and apoptosis [124]. In glioma cells, costunolide induced apoptosis in a reactive oxygen species (ROS)-dependent manner by increasing p53 abrogated telomerase activity [109]. In addition, costunolide decreased Nrf2 level in tumor cells [109,125] to dysregulate Nrf2-TERT oxidative defense in glioma cells [109]. In line with these in vitro data, the same authors found that Costunolide also reduced tumor burden in vivo using a glioma heterotypic xenograft mouse model [109]. Inhibition of Nrf2 also aids with increased sensitivity towards chemotherapeutic drugs such as TMZ [109]. Although information for TERT promoter mutations for this patient SANTB00442 is not available (Table 2), based on these previous antecedents, Costunolide may induce apoptosis of tumor cells through the inhibition of TERT, an effect that could have been further enhanced after chemotherapy and radiotherapy (i.e., by reduction of Nrf2 [109] in Stupp treated GBOs). To the best of our knowledge, this is the first study to show that Costunolide is effective at reducing cell viability of TMZ+radiation treated glioblastoma cells in an organoid model.

In general, we observed that 2D cultures were more sensitive to treatment compared to the matched 3D organoids (GBOs). Two of the major factors that could have contributed towards the ineffectiveness of some drugs in GBOs compared to G18-T cells are: (i) the access of the drugs to the cells; and (ii) the complex microenvironment of the GBOs. Cells in 2D culture are constantly bathed in the drug-containing media that can easily access all cells in culture, which may account for greater effectiveness of drug treatment observed in this model compared to the 3D model where cells in the core of the (non-vascularized) organoid [49] are not easily reached by the drug. Moreover, GBOs are known to retain tumor-associated macrophages/microglia, which play a major role in resistance to therapy through the induction of stemness [10,12,15]. Furthermore, the inability of the drugs to fully penetrate the GBO as well as potential hypoxia gradients within the GBOs [126,127] that can trigger the activation/expression of drug-resistant genes may have also contributed to the ineffectiveness of these drugs.

In summary, these results complementing the distinct advantages of patient-derived 2D and 3D models present a novel workflow for screening small groups of drugs with the potential for a more personalized approach in the treatment of recurrent glioblastoma. Moreover, it is possible to scale up this process further to include additional drugs that also shown promise in the clinical trial setup (e.g., regorafenib [128], and other Phase II-IV clinical trials drugs as recently reviewed by Cruz DaSilva et al. [129]) as well as drug candidates identified in large scale screenings using 2D cultures of patient-derived cells [130], which have not been yet tested in 3D GBO models.

## 4. Materials and Methods

### 4.1. Drug Library

We performed a literature review to identify promising targets for glioblastoma. In the first instance, we searched for drugs and targets that are being evaluated in Phase II–IV clinical trials for glioblastoma (https://clinicaltrials.gov, accessed in 1 April 2020) corresponding clinical trial data showing these drugs present some benefit to patients with less adverse effects. In addition, we also searched for FDA-approved drugs that target signaling pathways dysregulated in glioblastoma but which were not yet being investigated in clinical trials. Finally, we further identified recently discovered new targets for glioblastoma which are neither being used in clinical trials nor FDA approved. Following this review, we identified 64 drugs (i.e., excluding TMZ) that we used for our drug screening, of which 61 drugs were purchased from Selleck Chemicals, (Houston, TX, USA). Most of these 61 compounds were provided in 10mM stock concentrations and a few in 2 mM stock concentration diluted in either dimethyl sulfoxide (DMSO) (Cat# D2650, Sigma-Aldrich Pty Ltd, North Ryde BC, Australia) or water. Three of the 64 compounds [YAP/TAZ inhibitor-1 (Cat# HY-111429), Talampanel (Cat# HY-15079) and JR-AB2-011 (Cat# HY-122022)] were purchased from MedChemExpress, (Monmouth Junction, NJ, USA), in 5 mg powder format and diluted at 10 mM concentration in DMSO. TMZ (Cat#T2577) was purchased from Sigma-Aldrich Pty Ltd (North Ryde, BC, Australia) in 100 mg powder format and diluted to 10 mM stock concentration in DMSO.

### 4.2. Glioblastoma Cell Culture

FPW1 GSC was described previously [46] and patient-derived G18-T GSC was generated following the same protocol. GSCs were cultured in NSC medium containing 10 µg of recombinant human EGF; 10 µg of recombinant human FGF2; 10 mL of StemPro Neural Supplement (cat #A10509-01, StemPro NSC Serum-Free kit, ThermoFisher Scientific, Scoresby, Australia); 500 mL of serum-free Knockout DMEM/F-12 (Ref#12660-012, Gibco, ThermoFisher Scientific, Scoresby, VIC, Australia); and 5 mL GlutaMAX (cat#35050061, Gibco, ThermoFisher Scientific, Scoresby, VIC, Australia). Cells were cultured in T-75 culture flasks coated with Matrigel (Cat#FAL354234, Corning, Glendale, AZ, USA) diluted 1:100 in Dulbecco’s Phosphate Buffered Saline (DBPS1X, 4 mL, Gibco, ThermoFisher Scientific, Scoresby, VIC, Australia) for 30 min at 37 °C. Cells were grown at 37 °C in a 95% humidified atmosphere containing 5% CO_2_ until ~80–100% confluent. For passaging, cells were washed using 3 mL of DBPS1X and then treated with 4 mL of Accutase (cat# A6964, Sigma-Aldrich Pty Ltd, North Ryde BC, Australia) for 5–10 min (until cell dissociation is visible) at room temperature. Subsequently, 6 mL of pre-warmed serum-free Knockout DMEM/F-12 was added to the flask and the cells were resuspended and centrifuged (500× *g*, 5 min). Following centrifugation, cell pellets were resuspended into fresh 2 mL StemPro NSC medium and from the cell suspension, the appropriate number of cells were transferred to either fresh Matrigel-coated T-75 flask or 384-well plates.

### 4.3. Seeding of Tumor Cells into 384-Well Plates

First, 384-well plates with optically clear polymer bottom (Ref# 142762, ThermoFisher Scientific, Scoresby, VIC, Australia) were pre-coated with 50 μL/well of 1:100 Matrigel:DPBS solution using Eppendorf Single Channel Repetitive pipette and incubated at 37 °C for at least 30 min and then aspirated before seeding patient-derived GSC cells. A suspension of GSC cells (FWP1 or G18-T) prepared by treatment with Accutase as described above was resuspended in 25 mL of StemPro NSC medium. Cell counting on this suspension was performed using the Scepter™ 2.0 Cell Counter (Millipore, Bayswater, Australia). Cell concentration was then adjusted to a concentration of 1.848 × 10^5^ cells/mL and cells were transferred into 384-well plates (50 μL/well, ~10,000 cells/well). The plates were incubated in a humidified environment at 37 °C and 5% CO_2_ for one day before being subject to treatment with different drugs.

### 4.4. Serial Drug Dilutions and Addition of Drugs to Cells Using Automated Liquid Handler Robot

Approximately 24 h after seeding, various drug concentrations (500, 100, 50, 10, 1, 0.5, 0.1 and 0 µM) diluted in normal culture media were transferred to G18-T or FWP1 cells plated in 384-well plates by utilizing the OT-2 liquid handler robot and associated software (Opentrons, Brooklin, NY, USA). Custom labware templates for 96-well plate (cat#P96- 1.5H-N, Cellvis, Mountain View, CA, USA), 384-well plate and reservoir were created using the measurements provided by the manufacturer for each item using the Opentrons labware creator. Moreover, a protocol script was created in Python that enable transfer of the drugs to tumor cells cultured in 384-well plates, incorporating the GEN1 single channel p300 pipette and a temperature module to maintain cells at 37 °C, while drugs were transferred to each plate. Before running the protocol, 0.5 mM (500 µM) of 67 drugs were individually diluted in StemPro NSC medium in the wells of row A of seven 96-well plates from the 10 or 2 mM stock concentration of each drugs. Then, the protocol was run through the steps as detailed in Figure S2, and, after drug addition, multiwell plates were incubated in a humidified environment at 37 °C and 5% CO_2_ for 72 h before viability measurements.

### 4.5. Cell Viability Assay

Approximately 72 h after the addition of drugs to cells in 384-well plates, CellTiter-Glo^®^ Luminescent Cell Viability Assay (Cat# G7570, Promega Australia, Alexandria, Australia) was performed to assess cell viability though the detection of cellular metabolic adenosine triphosphate (ATP) levels. For this, 30 µL of CellTiter-Glo^®^ 2.0 reagent were transferred to each well of the 384-well plates and incubated at room temperature on an orbital shaker for 2 min at 40 RPM to induce cell lysis. Ten minutes later, luminescence signal was recorded using the FLUOstar Omega (BMG LABTECH, Pty. Ltd, Mornington, Australia) microplate reader. The luminescence data were then analyzed using MATLAB (Supplementary Material) and IC_50_ for each drug was calculated using dose vs. response curves by unconstrained non-linear regression (Equation (1)) using Prism 8. IC50 heatmap was generated using Morpheus (https://software.broadinstitute.org/morpheus/#, accessed in 1 November 2020).

(1) $$\mathrm{Viability}\;\left(a.u\right)={\mathrm{Resistant}\;\mathrm{cells}}^{’}\;\mathrm{viability}+{\;\mathrm{Sensitive}\;\mathrm{cells}}^{’}\mathrm{viability}\;\times \frac{1}{\left[\frac{Drug\;concentration}{IC50}\right]}$$

### 4.6. Generation of Glioblastoma Organoids (GBOs)

The GBOs used in this project were generated and cultured from a patient-derived tumor sample from the SANTB using the method described initially by Jacob and collaborators [49], whose experimental procedure is explained in full detail in a follow-up protocol paper [48].

### 4.7. TMZ+Radiation Treatment (Stupp Protocol) “In a Dish”

G18-T cells were seeded into 2 wells of 6-well plates which were pre-coated with Matrigel diluted 1:100 in PBS and after 24 h, the plate received 2 Gy of irradiation followed by 2 mL/well of 50 µM TMZ in StemPro NSC medium. On alternating days, the media was replaced with 2mL of fresh media containing the same TMZ concentration. An equivalent 2 mL of medium without TMZ was delivered to each control well. 

During the same time, 5 GBOs per well were transferred into 2 wells each of treatment and control 6-well plates. As with the cells, the treatment plate received two Gy of irradiation followed by 50 µM concentration of TMZ. Every alternative day, 3 mL of the media (of a total of 4 mL) was replaced with 3 mL of fresh media containing the same TMZ concentration. For the control plate, 3 mL of fresh GBO medium without TMZ was delivered to each well. This treatment was performed every other day over a 10-day period. 

The treatment plates had received a total of 10 Gy irradiation treatment by the end of day 10. Brightfield images of all control and treatment plates were taken every two-days using the IN Cell Analyzer 2200 (Cytiva, North Ryde, NSW, Australia). After 10 days of treatment, G18-T cell and GBO culture was continued in their normal growth medium for approximately 2 weeks before treatment with selected drugs.

### 4.8. Treatment with Selected Drugs

After 2 weeks, both control and “Stupp” treated G18-T cells were detached from the 6-well plates and seeded into two separate 384-well plates (18 wells per plate) pre-coated with Matrigel. The plates were incubated in a humidified environment at 37 °C and 5% CO_2_. Approximately 24 h later, 50 µM concentration of Vismodegib, Disulfiram, Parthenolide, Omipalisib and Costunolide were prepared in NSC medium. Then, 30 µL of each drug or Stempro NSC medium (control) were delivered to cells in 384-well plates in triplicates. After 5 days, a cell viability assay was performed as described above and data obtained was analyzed using GraphPad Prism v8.

For GBOs, 6 control and 6 previously treated GBOs were transferred to separate 24-well plates. Subsequently, 50 µM concentration of Vismodegib, Disulfiram, Parthenolide, Omipalisib and Costunolide were prepared in 5 mL GBO medium and were delivered to both control and treated GBOs. On alternating days, the medium was replaced with 2 mL of fresh media containing the same drug concentration for each condition. This treatment was performed for over 10 days. Brightfield images of both control and treatment plates were taken on the days when there was no treatment done using the IN Cell Analyzer 2200 (Cytiva, North Ryde, Australia).

### 4.9. Statistical Analysis

Data from the cell viability assay for G18-T cells were analyzed using either *t*-test or two-way analysis of variance (ANOVA) corrected for multiple comparisons as detailed in the corresponding figure legend. Statistical analyses were performed using GraphPad Prism v8.

### 4.10. Artwork

All figures were created using Adobe Illustrator 2020, except Figure 1 and Figure 3 that were created with Biorender.com (https://biorender.com/, accessed in 1 January 2021).

## 5. Conclusions

The findings from this study reflect the high level of inter- and intra-tumoral heterogeneity present in glioblastoma, as the tested drugs had selective responses in different patient-derived in vitro models. Treatment of patient-derived glioblastoma cells in a 2D model demonstrated increased efficacy when drugs that exhibited the most selective responses were combined with TMZ and radiation compared to single drug therapy. However, only the TERT inhibitor Costunolide was effective on the Stupp resistant 3D GBO model, reflecting the contribution of tumor microenvironment to therapy resistance. These results highlight the potential role of TERT in phenotypic switching in glioblastoma as a key mechanism to develop resistance to therapy. Future studies should consider this approach to identify potential targets to overcome resistance to the current standard of care for glioblastoma.

## Figures and Tables

**Table 1:** List of selected drugs with their respective molecular target, signaling pathway and clinical status.

Drug Name	Target	Pathway	Phase II-IV Clinical Trial	FDA-Approved	Future Potential	Ref.
			34 Compounds	19 Compounds 5 (in Trials) 14 (not in Trials)	16 Compounds	
gossypol-acetic acid	5α-reductase 1 and 3α-hydroxysteroid dehydrogenase	Metabolism	−	−	+	[52]
AZD5363	Akt	PI3K/Akt/mTOR	+	−	−	[53]
Disulfiram	ALDH	Metabolism	+	−	−	[54]
Talampanel	AMPA		+	−	−	[55]
HA14-1	Bcl-2	Apoptosis	−	−	+	[56]
ABT-263	Bcl2/Bcl-XL	Apoptosis	−	−	+	[57]
Dasatinib	Bcr-Abl, c-Kit, Src	Angiogenesis	+	−	−	[58]
Dabrafenib (GSK2118436)	B-Raf	MAPK	+	−	−	[59]
Sorafenib	C/B-Raf	MAPK	+	+	−	[60]
Palbociclib (PD-0332991) HCl	CDK	Cell cycle	+	−	−	[61]
Abemaciclib	CDK4/6	Cell cycle	+	−	−	[62]
Celecoxib	COX-2	Neuronal signaling	+	−	−	[63]
Pexidartinib (PLX3397)	CSF-1R, c-Kit	Growth factor signaling	+	−	−	[64]
Plerixafor (AMD3100)	CXCR4	GPCR and G Protein	−	+	−	[65]
Rucaparib (AG-014699)	PARP	DNA damage	+	−	−	[66]
Thioguanine	DNA/RNA synthesis	Epigenetics	−	+	−	[67]
RITA (NSC 652287)	E3 Ligase, p53	Apoptosis	−	−	+	[68]
Osimertinib (AZD9291)	EGFR	Growth factor signaling	−	+	−	[69]
Cetuximab	EGFR	Growth factor signaling	+	−	−	[70]
Tazemetostat (EPZ-6438)	EZH2	Epigenetics	+	−	−	[71]
Tipifarnib	farnesyltransferase	Metabolism	+	−	−	[72]
Pacritinib (SB1518)	FLT3, JAK	JAK/STAT	+	−	−	[73]
Gilteritinib (ASP2215)	FLT3, TAM Receptor	Growth factor signaling	−	+	−	[74]
CID 1375606	GPR27	G Protein	−	−	+	[75]
Trichostatin A	HDAC I and II	Metabolism	+	−	−	[76]
Vismodegib (GDC-0449)	Hedgehog/Smoothened	Stem Cells and Wnt signaling	+	+	−	[77]
Neratinib (HKI-272)	HER2	Growth factor signaling	−	+	−	[78]
Crizotinib (PF-02341066)	HGFR, c-Met	Growth factor signaling	−	+	−	[79]
2-Methoxyestradiol (2-MeOE2)	HIF	Angiogenesis	+	−	−	[80]
Embelin	IAP	Apoptosis	−	+	−	[81]
Ivosidenib (AG-120)	IDH1	Metabolism	−	+	−	[82]
Enasidenib (AG-221)	IDH2	Metabolism	−	+	−	[83]
Indoximod (NLG-8189)	IDO	Metabolism	+	−	−	[84]
Epacadostat (INCB024360)	IDO1	Metabolism	−	+	−	[85]
Mycophenolate Mofetil	IMPDH	Metabolism	−	+	−	[86]
Cilengitide trifluoroacetate	Integrin	Cytoskeletal Signaling	+	−	−	[87]
SP600125	JNK	MAPK	−	−	+	[88]
Trametinib (GSK1120212)	MEK	MAPK	+	−	−	[89]
Cobimetinib (GDC-0973, RG7420)	MEK	MAPK	+	−	−	[90]
Selumetinib (AZD6244)	MEK1/2	MAPK	+	−	−	[91]
U0126-EtOH	MEK1/2	MAPK	−	−	+	[92]
Azacitidine	MGMT	DNA Damage	−	+	−	[93]
Everolimus (RAD001)	mTOR	PI3K/Akt/mTOR	+	+	−	[94]
AZD8055	mTORC1	PI3K/Akt/mTOR	+	−	−	[95]
JR-AB2-011	mTORC2	PI3K/Akt/mTOR	−	−	+	[96]
Bortezomib (PS-341)	NF-κB	Proteases	+	−	−	[97]
Parthenolide	HDAC, IKK-β, NF-κB	NF-κB	−	+	−	[98]
Isotretinoin	others	others	+	−	−	
Oroxin A	Others	Others	−	−	+	
Oroxin B	Others	Cancer	−	−	+	[99]
Nutlin-3	P53, Mdm2	Apoptosis	+	−	−	[100]
Veliparib (ABT-888)	PARP	DNA Damage	+	−	−	[101]
Olaparib (AZD2281, Ku-0059436)	PARP	DNA Damage	+	−	−	[102]
Imatinib Mesylate (STI571)	PDGFR	Growth factor signaling	+	+	−	[103]
Omipalisib (GSK2126458, GSK458)	PI3K	PI3K/Akt/mTOR	−	−	+	[104]
Duvelisib (IPI-145, INK1197)	PI3K	Angiogenesis	−	+	−	[105]
S3I-201	STAT	JAK/STAT	−	−	+	[106]
Cryptotanshinone	STAT	JAK/STAT	−	−	+	[107]
WP1066	STAT3	JAK/STAT	−	−	+	[108]
Costunolide	TERT	DNA Damage	−	−	+	[109]
O6-Benzylguanine	Transferase/ AGT	Metabolism	+	−	−	[110]
Pazopanib	Tyrosine kinase	Growth factor signaling	+	+	−	[111]
Cediranib (AZD2171)	VEGFR	Growth factor signaling	+	−	−	[112]
Yap/TAZ inhibitor-1	YAP/TAZ	Hippo Pathway	−	−	+	[113]

**Table 2:** Patient demographics corresponding to in vitro models used in this study.

Patient	Age (Years)	Gender	Tumor Type	Tumor Site	Survival (Days)	IDH Status	MGMT Status	TERT Prom. Mutations
FPW1 [46,118]	68	Male	Primary glioblastoma	Right temporal	242	WT	unmethylated	none
SANTB00442 *	49	Male	Primary glioblastoma	Left frontal	99	WT.	not available	Not available

* G18T cells and GBOs were derived from tumor tissue resected from this patient.

**Table 3:** Targets for “Group 3” drugs with selective effect in each cell line.

G18-T Cells		FPW1 Cells	
Drug Name	Target	Drug Name	Target
Pazopanib	c-kit, PDGFR, VEGFR	AZD5363	Akt
Disuliram	ALDH	Pexidartinib	CSF-1R, c-Kit
RITA	E3 ligase, p53	Dafrafenib	Raf
Oroxin B	ER	S31-201	STAT
2-methoxyestradiol	GPR30	Temozolomide	DNA damage
Vismodegib	Hh/GLI		
Costunolide	hTERT		
Trametinib	MEK		
AZD8055	mTOR		
Partenolide	HDAC, IKK-β, NF-κB		
Imatinib Mesylate	PDGFR		
Omipalisib	PI3K/mTOR		
WP1066	Stat3		

## Data Availability

Computational scripts for visualization of bioluminescent data for analysis of drug response in different plates are provided as Supplementary Materials. Computational scripts for operation of Opentrons OT-2 robot for serial dilutions (Figure S2) are available from the corresponding author on reasonable request.

## Supplementary Materials

The following are available online at https://www.mdpi.com/article/10.3390/ijms22094322/s1.

## Abbreviations

2D	two dimensional
3D	three dimensional
ATP	adenosine triphosphate
CAR-T	chimeric antigen receptor T cells
CSC	cancer stem cell
DPBS	Dulbecco’s Phosphate Buffered Saline
EGFR	epidermal growth factor receptor
FDA	U.S. Food & Drug Administration
GBO	glioblastoma explant organoid
GSC	glioma stem cell
IC50	half maximal inhibitory concentration
IDH1	isocitrate dehydrogenase 1
MET	mesenchymal to epithelial transition
MGMT	O[6]-methylguanine-DNA methyltransferase
NF1	neurofibromatosis 1
PDGFRA	Platelet-derived growth factor receptor A
PTEN	Phosphatase and tensin homolog
RTK	receptor tyrosine kinases
SANTB	South Australian Neurological Tumour Bank
scRNAseq	single-cell RNA sequencing
NSC medium	StemPro Neural Stem Cell serum-free medium
TAM	tumor-associated macrophages
hTERT	Human Telomerase reverse transcriptase
TERT	Telomerase reverse transcriptase
TMZ	temozolomide
VEGF	vascular endothelial growth factor
