# Supplementary figures and images for: A Drug Screening Pipeline Using 2D and 3D Patient-Derived In Vitro Models for Pre-Clinical Analysis of Therapy Response in Glioblastoma
Source: Int J Mol Sci. 2021 Apr 21;22(9):4322. doi: 10.3390/ijms22094322 (PMC8122466; doi:10.3390/ijms22094322)

# Supplementary Figure 1

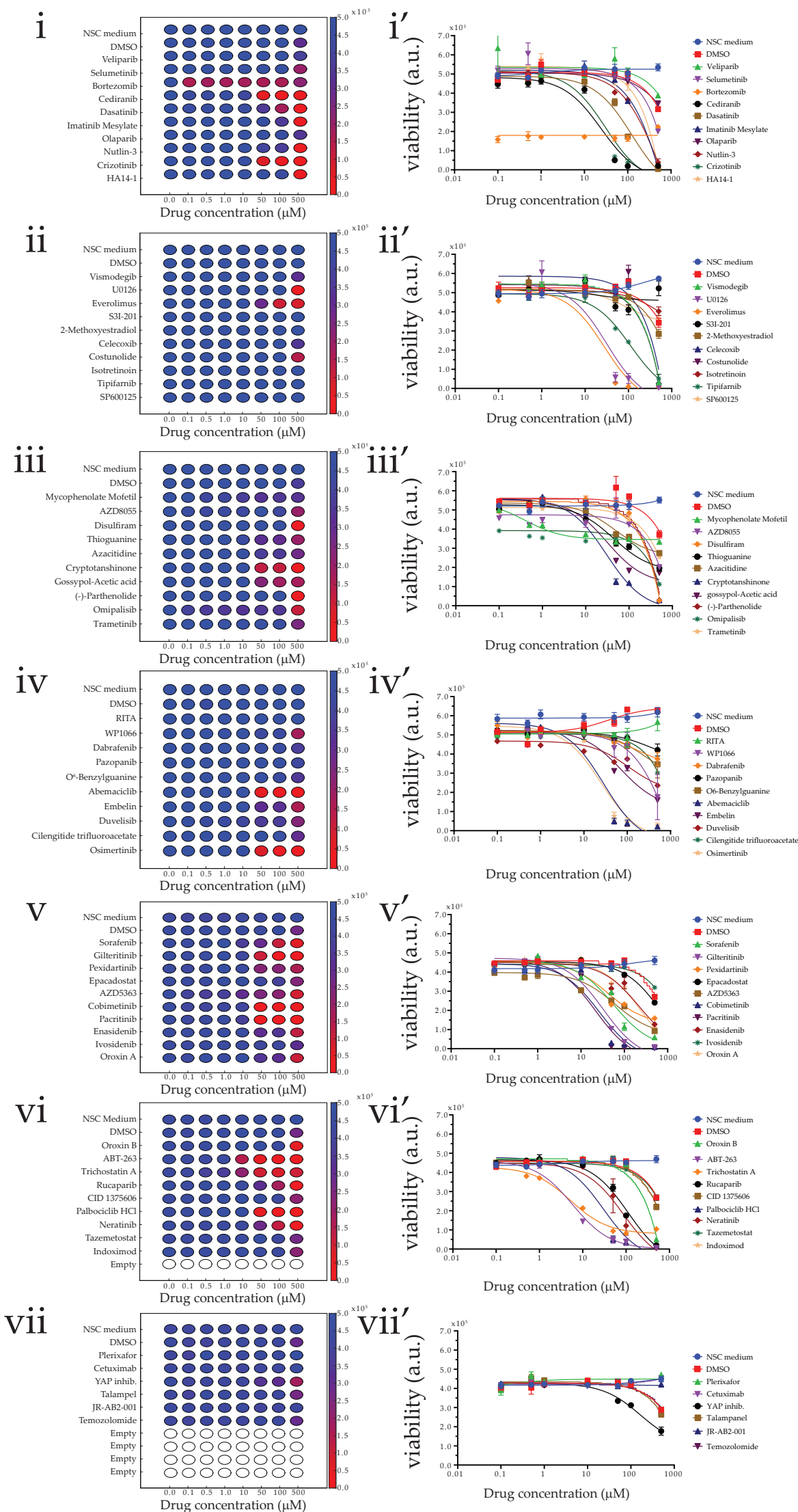

Supplement: Supplementary file 1 [file ijms-22-04322-s001.zip › Lenin et al_Supplementary_Figure 1.pdf]
